# Sulforaphane, quercetin and catechins complement each other in elimination of advanced pancreatic cancer by miR-let-7 induction and K-ras inhibition

**DOI:** 10.3892/ijo.2014.2539

**Published:** 2014-07-08

**Authors:** MAHESH APPARI, KAMESH R. BABU, ADAM KACZOROWSKI, WOLFGANG GROSS, INGRID HERR

**Affiliations:** 1 Molecular Oncosurgery, University Clinic of Heidelberg and German Cancer Research Center (DKFZ), Heidelberg, Germany; 2 Department of General and Transplantation Surgery, University of Heidelberg, Heidelberg, Germany; 3 Department of Pediatric Oncology, Hematology, Immunology and Pulmonology, University of Heidelberg, Heidelberg, Germany

**Keywords:** bioactive agents, cancer stem cells, miRNA signaling, pancreatic cancer

## Abstract

Pancreatic ductal adenocarcinoma (PDA) has the worst prognosis of all malignancies, and current therapeutic options do not target cancer stem cells (CSCs), which may be the reason for the extreme aggressiveness. The dietary agents sulforaphane and quercetin enriched e.g., in broccoli, and the main and best studied green tea catechin EGCG hold promise as anti-CSC agents in PDA. We examined the efficacy of additional catechins and the combination of these bioactive agents to stem cell features and miRNA signaling. Two established and one primary PDA cell line and non-malignant pancreatic ductal cells were used. Whereas each agent strongly inhibited colony formation, the catechins ECG and CG were more effective than EGCG. A mixture of green tea catechins (GTCs) significantly inhibited viability, migration, expression of MMP-2 and -9, ALDH1 activity, colony and spheroid formation and induced apoptosis, but the combination of GTCs with sulforaphane or quercetin was superior. Following treatment with bioactive agents, the expression of miR-let7-a was specifically induced in cancer cells but not in normal cells and it was associated with K-ras inhibition. These data demonstrate that sulforaphane, quercetin and GTC complement each other in inhibition of PDA progression by induction of miR-let7-a and inhibition of K-ras.

## Introduction

Pancreatic ductal adenocarcinoma (PDA) is one of the most aggressive malignancies usually diagnosed in an advanced state, with an extensive local invasion, early systemic dissemination and marked resistance to chemo- and radiotherapy (1). A frequently dysregulated gene is K-ras, which is overexpressed in more than 90% of patients suffering from PDA (2). The current treatment options for PDA provide a 5-year survival rate of only 5%. Cancer stem cells (CSCs) are made responsible for the pronounced therapy resistance and early progression of PDA, because this small subpopulation within the tumor mass is thought to survive conventional cytotoxic therapy due to defense and survival mechanisms (3,4). CSCs are believed to possess self-renewal potential and the ability to divide asymmetrically, whereby a mother cell generates a stem cell and a progenitor cell, which undergoes clonal expansion and then terminally differentiates (5–7). Because current therapeutics do not target CSCs, new therapeutic options are urgently needed.

Several epidemiological cancer and nutrition studies provide new therapeutic perspectives, because they show that the risk to develop cancer and the risk of cancer progression correlates with a defined dietary pattern. One well-designed large population-based case-control study found that frequent consumption of cruciferous vegetables, three and more servings/week of e.g., broccoli, cauliflower, cabbage, radish, horseradish, cress, arugula, mustard, canola and others, had an about 50% risk reduction to develop pancreatic cancer (8). Another prospective epidemiological study found that the high consumption of cruciferous vegetables and especially of broccoli and cauliflower was associated with a 50 % reduction of the relative risk of developing metastasis in patients with prostate cancer (9). Similarly, a population-based case-control study conducted in urban Shanghai, China, found that regular green tea drinking was associated with a 32% reduction of pancreatic cancer risk in women (10). Well examined bioactive substances from broccoli, cauliflower and green tea involve the isothiocyanate sulforaphane (11), the polyphenol family of catechins (12) and the polyphenol quercetin, which is one of the most abundant flavonoids found in many fruits and vegetables, such as broccoli, apples, onions and berries (13).

Our recent *in vitro* data and mouse experiments suggest that sulforaphane eliminates pancreatic CSCs by inhibition of NF-κB activity and self-renewal potential and thereby sensitizes the cells to apoptosis induction (14–16). After publication of these results many patients contacted us, and we recommend a balanced plant-based diet enriched in cruciferous vegetables. We do not recommend a focus to cruciferous vegetables or broccoli sprout supplements, because in the meanwhile also other plant ingredients with anti-CSC activity have been identified (17). In particular, quercetin and epigallocatechin-3-gallate (EGCG) have been described to target pancreatic CSCs (18,19). Other dietary compounds with experimental proven direct or indirect effects on Wnt/β-catenin, hedgehog and Notch self-renewal pathways include the turmeric spice component curcumin, soy isoflavone, resveratrol, lycopene, piperine and vitamin D3 (17). There may still be several other plant substances with similar activity. Therefore, the high intake of a balanced mixture of a plant-based diet with proven activity toward CSCs may be superior to the intake of supplements with isolated substances.

Recently, the indole 3,3′-diindolylmethane (cruciferous vegetables), the polyphenol genistein (soy) and an analogue of the natural phenol curcumin (turmeric) have been demonstrated to inhibit the growth of pancreatic and prostate cancer by upregulation of the micro RNA (miR)-let-7 (20–22). The miR-let-7 (from ‘lethal’) is one of the first identified miRNAs, due to its role in terminally differentiation of *C. elegans* seam cells (23). A function of miR-let-7 in human cancer was detected, and expression levels of let-7 members are significantly low in human cancers and CSCs. The major function of let-7 is to promote the terminal differentiation in development and tumor suppression (24). Let-7 has been demonstrated to be a direct regulator of K-ras expression in human cells (25). In lung cancer patient samples, expression of K-ras and let-7 showed reciprocal patterns of low let-7 and high K-ras in cancerous cells, but high let-7 and low K-ras in normal cells (25). In many cancer types, downregulation of miR-let-7 is associated with a poor outcome for patients (26).

In the present study we asked if green tea-derived catechins in general may harbor anti-CSC activity, if the combination with quercetin and sulforaphane may be superior and if the upregulation of miR-let-7 and the downregulation of K-ras are involved. We demonstrate that epicatechin-3-gallate (ECG) and catechin gallate (CG) are as potent as EGCG in inhibition of colony-formation. Furthermore, sulforaphane, quercetin and a natural mixture of the complete set of green tea catechins (GTC) complemented each other in induction of apoptosis and inhibition of self-renewal potential, migration and expression of the matrix metalloproteinases MMP-9 and MMP-2. Most importantly, these bioactive agents led to induction of miR-let-7 and inhibition of its target gene K-ras with strongest effects after combination.

## Materials and methods

### Established cell lines and primary cells

The human established PDA cell lines BxPc-3 and MIA-PaCa2 and human hTERT-HPNE immortalized pancreatic duct cells CRL-1097 were obtained from the American Type Culture Collection (Manassas, VA, USA). The primary human PDA cells PaCaDD-183 were isolated from a patient PDA tissue as described (27). MIA-PaCa2 and BxPc-3 cells were cultured in DMEM (PAA, Pasching, Austria) supplemented with 10% heat-inactivated FCS (Sigma, Deisenhofen, Germany) and 25 mmol/l HEPES (PAA). Cells were authenticated throughout the culture by the typical morphology. To maintain authenticity of the cell lines, frozen stocks were prepared from initial stocks, and every three months a new frozen stock was used for the experiments. The authenticity of established cell lines was certified in April 2013. Mycoplasma-negative cultures were ensured by monthly testing.

### Treatment of cells

Epigallocatechin gallate (EGCG), epicatechin gallate (ECG), and catechin gallate (CG) (Sigma) were diluted in water to a 50-mM stock solution. Green tea extract (GTC) containing 588 mg polyphenols/600 mg, from which 50% are EGCG (Dr. Loges + Co. GmbH, Winsen, Germany) were diluted in DMSO to a 300-mM stock solution. Quercetin (Sigma) was diluted in DMSO to a 200-mM stock solution and DL-sulforaphane (Sigma) was diluted in EtOH to a 100-mM stock solution. The final concentration of the solvents in cell culture assays was 1:1000 or higher.

### Viability assay

Viability was measured using 3-(4,5-dimethylthiazol- 2-yl)-2,5-diphenyltetrazolium bromide (MTT) as described previously (14).

### Apoptosis measurement

Cells were stained with fluorescein isothiocyanate (FITC)-conjugated Annexin V (BD Biosciences, Heidelberg, Germany) and externalization of phosphatidylserine was identified by flow cytometry (FACScan, BD Biosciences). DNA fragmentation was detected by staining of fragmented DNA with propidium iodide buffer according to Nicoletti *et al* (28).

### Detection of ALDH1 activity

A total of 2.5 μl of Aldefluor substrate (Aldagen, Durham, USA) was added to 1×10^6^ cells in 500 μl of assay buffer and incubated for 60 min at room temperature. As a negative control diethylamino-benzaldehyde (DEAB) was added to the cells. ALDH1 activity was measured by flow cytometry (FACScan, BD Biosciences).

### Colony-forming assay

Seventy-two hours after treatment cells were seeded in complete medium in 6-well tissue culture plates (TPP) and colony-forming assays were performed as described previously (14).

### Spheroid assay

For formation of spheroids, cells were cultured in NeuroCult NS-A basal serum-free medium (human) (StemCell Technologies, Vancouver, Canada) supplemented with 2 μg/ml heparin (StemCell Technologies), 20 ng/ml hEGF (R&D Systems, Wiesbaden-Nordenstadt, Germany), 10 ng/ml hFGF-b (PeproTech, Hamburg, Germany) and NeuroCult NS-A Proliferation Supplements (StemCell Technologies). Cells were seeded at low densities (2×10^2^ to 1×10^3^ cells/ml) in 12-well low-adhesion plates (1 ml/well). Five days later, spheroid formation was visible and spheroids were treated. Forty-eight hours after treatment spheroids were dissociated by vigorous pipetting up and down, followed by evaluation of the number of trypan-blue negative cells, and re-seeding as described above (1st generation). Upon formation of spheroids, cells were re-treated with natural substances and the number of viable cells was evaluated by counting trypan blue-negative cells (2nd generation).

### Western blot analysis

Proteins were isolated, and western blot analysis was performed as described previously (14). The following antibodies were used: rabbit polyclonal Ab against human ras (Cell Signaling Technology, Danvers, MA, USA) - this antibody detects endogenous levels of total K-ras, H-ras and N-ras and it may cross-react with R-ras and M-ras. Mouse mAb against human β-actin was from Sigma.

### Detection of MMP-2, MMP-9 and K-ras mRNA expression

Total RNA was extracted with the RNeasy Mini Kit (Qiagen, Hilden, Germany) according to the manufacturer’s instructions. cDNA was synthesized with the iScript™ cDNA Synthesis kit (Bio-Rad, Munich, Germany). qRT-PCR was performed by using reagents and a LightCycler^®^-PCR machine (Roche Diagnostics GmbH, Mannheim, Germany) as described recently (29). Specific primer pairs (MWG Biotech, Ebersberg, Germany) were: β-actin 5′-TGACGGGGTCACCCACACTGTG CCCATCTA-3′ (sense), 5′-CTAGAAGCATTTGCGGTGGAC GATG-3′ (antisense); MMP-9: 5′-GACCTCAAGTGGCACC ACCA-3′ (sense), 5′-GTGGTACTGCACCAGGGCAA-3′ (antisense); MMP-2 5′-AGTCTGAAGAGCGTGAAG-3′ (sense), 5′-CCAGGTAGGAGTGAGAATG-3′ (antisense), K-ras 5′-AT TCCTTTTATTGAAACATCAGCA-3′ (sense), 5′-TCGGAT CTCCCTCACCAAT-3′ (antisense). The PCR conditions were for MMP-2 and K-ras: 95′C for 20 sec followed by 58°C for 30 sec; MMP-9: 95°C for 20 sec followed by 60°C for 30 sec. Fold changes of target gene expression relative to β-actin were calculated using the 2-^ΔΔCt^ method.

### Detection of microRNA expression

MicroRNA was extracted with the mirVana miRNA isolation kit (Applied Biosystems/Life technologies, Darmstadt, Germany) according to the manufacturer’s instructions. Total RNA containing miRNA was synthesized to cDNA with miScript II RT kit (Qiagen). Quantification of miRNA was performed by using the miScript SYBR-Green PCR kit (Qiagen) and a LightCycler-PCR (Roche Diagnostics GmbH). Transcripts obtained with U6 primers were used for normalization. The sense sequence of miR-let-7a was 5′-TGAGGTAGTAGGTTGTATAGTTGG-3′ (MWG Biotech); A universal antisense primer and the U6 control primers were provided in the miScript SYBR-Green PCR kit. The PCR conditions were 95°C for 15 sec, 60°C for 20 sec. Fold changes of miR-let-7a gene expression relative to U6 were calculated using the 2-^ΔΔCt^ method.

### Transwell migration assay

To analyze the cell invasive potential we used a standard transwell assay. Transwell polycarbonate filters of 8-μm pore size (Corning, Inc., Lowell, MA) were used. FCS was used as a chemo-attractant with DMEM in the lower compartment. Sulforaphane, quercetin and catechin pre-treated (18 h) or untreated cells were seeded at a concentration of 10^5^ cells/cm^2^ in 24-well plates. The cells in boyden chambers were incubated at 37°C and 5% CO_2_. After 24 h the number of transmigrated cells was counted. The percentage of transmigrated cells was normalized to the percentage of cell vitality evaluated by the MTT assay at the end-point of the experiment.

### Statistical analysis

The quantitative data are presented as the mean ± SD. The groups were compared using the nonparametric Mann-Whitney test. We report point-wise P-values not adjusted for multiple comparisons since this project is exploratory in nature. The control was compared to the single treatment groups and the single treatment groups were compared to the double treatment groups. ^*^P<0.05 and ^**^P<0.01 were deemed to be statistically significant.

## Results

### The combination of dietary agents is superior in reducing the self-renewal potential compared to single agents

**Figure 1:**
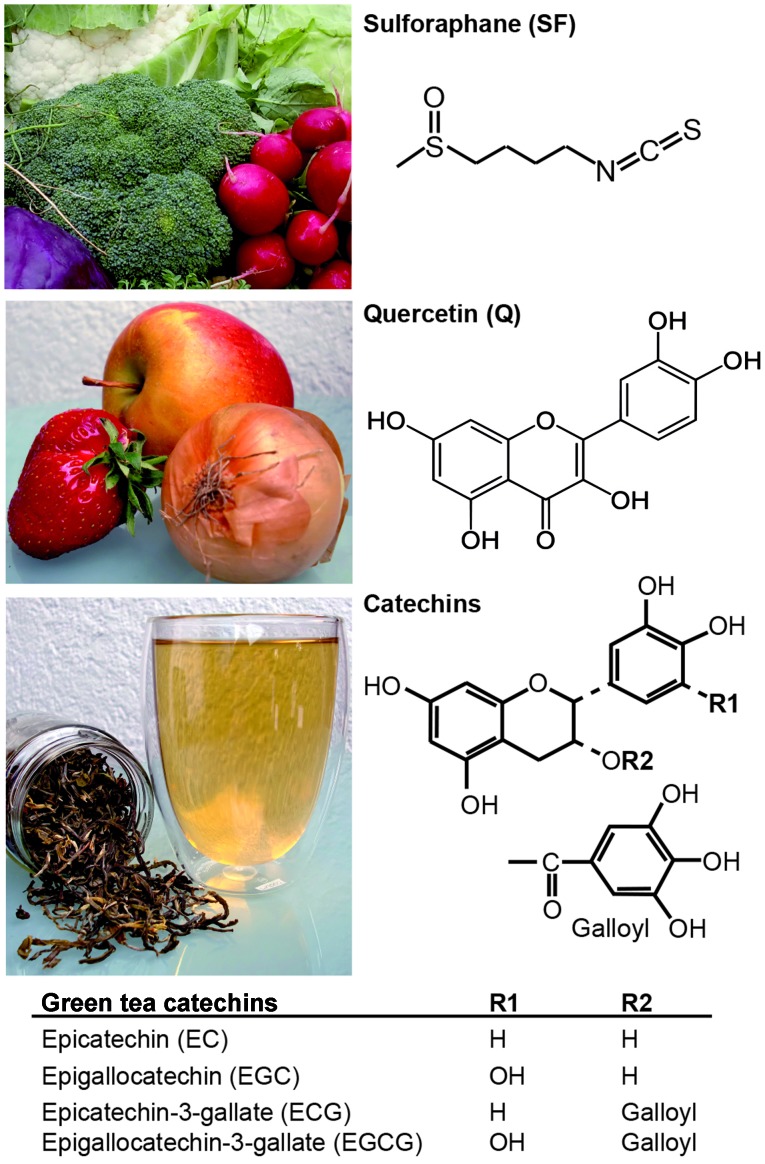
Chemical formulas of sulforaphane, quercetin and green tea catechins and photographs of fruits and vegetables harboring these dietary agents.

Recently, sulforaphane, quercetin and EGCG (Fig. 1) were described to target pancreatic CSC features (14,18,29,30). We asked if other green tea catechins may have similar activity and if the combination of bioactive agents may enhance the efficacy. The human established PDA cell lines MIA-PaCa2 with high CSC features and BxPc-3 with low CSC features served as model cell lines (compare Table I).

**Figure 2:**
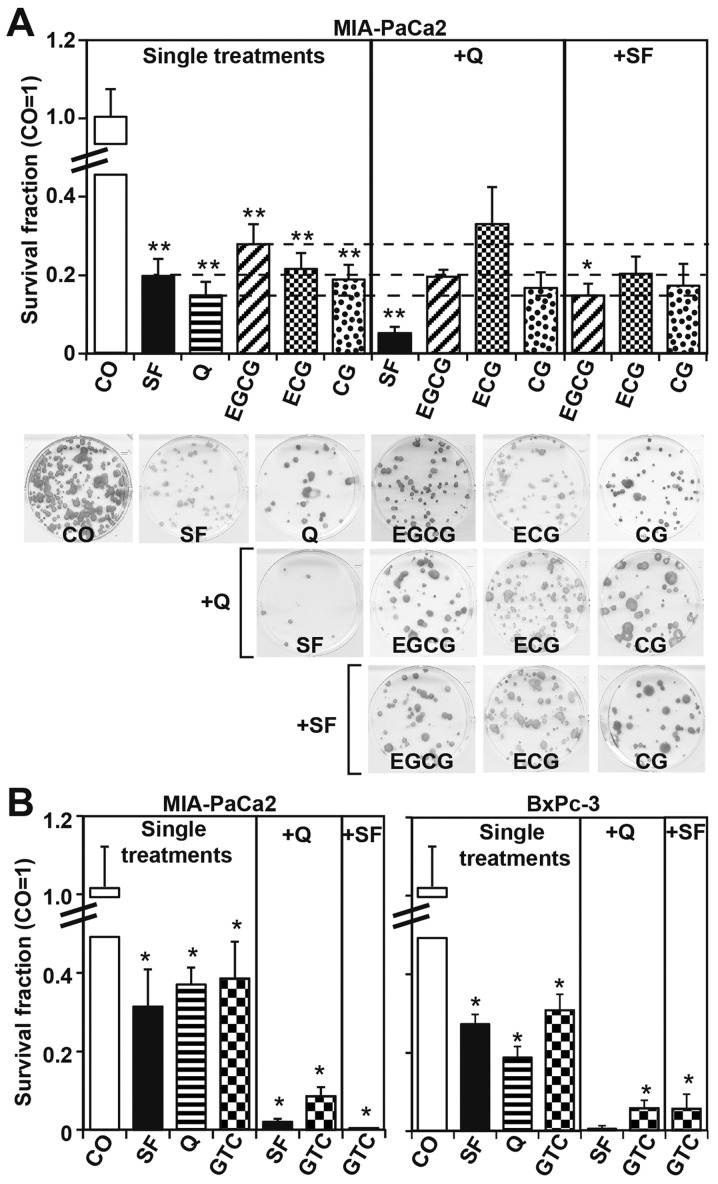
Combinations of dietary agents are superior in reducing clonogenicity compared to single agents. (A) MIA-PaCa2 cells were left untreated or were treated with 10 μM sulforaphane (SF), 200 mM quercetin (Q), 40 μM epigallocatechin gallate (EGCG), 40 μM epicatechin gallate (ECG) or 40 μM catechin gallate (CG) or combinations thereof as indicated. Seventy-two hours later the cells were trypsinized and equal numbers of living cells per group were re-plated to 6-well plates (200 cells/well). Cells were grown without a change of medium for 2 weeks, followed by fixing and Coomassie-staining of colonies and determination of the number of colonies consisting of at least 50 cells. The plating efficiency was calculated using the formula: ‘number of colonies/number of seeded cells’ and is presented as survival fraction after setting the control to 1. Samples of the colonies are shown. (B) MIA-PaCa2 and BxPc-3 cells were treated as described above, but the single green tea catechins were replaced by a 40-μM green tea extract (GTC) containing all green tea catechins together. Seventy-two hours later 200 MIA-PaCa2 cells or 2000 BxPc-3 cells were re-plated per well of 6-well plates, followed by evaluation of colony-formation 2 weeks later. Means of three independent experiments ± SD are shown. The significance of each single treatment compared to the control and the significance of each single treatment compared to the respective double treatment was analyzed by the Student’s t-test and is marked by an asterisk. ^*^P<0.05, ^**^P<0.01.

**Figure 3:**
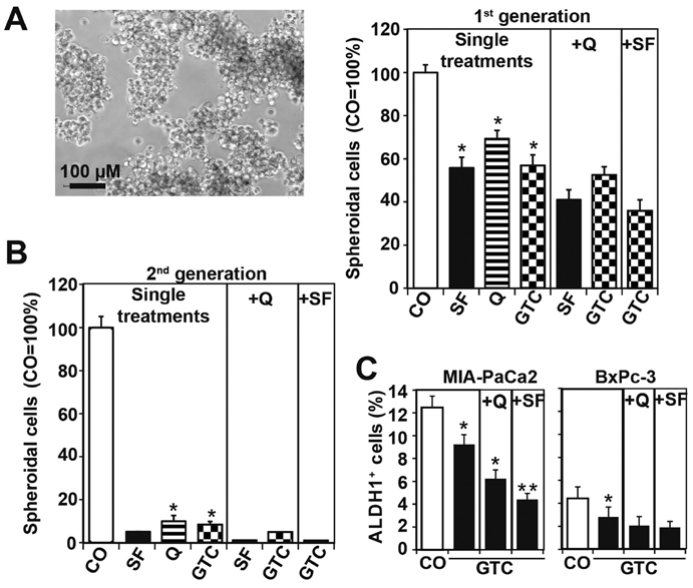
Combinations of dietary agents are superior in reducing spheroid formation compared to single agents. (A) MIA-PaCa2 cells were seeded at clonal density in low-adhesion plates for spheroid formation. Three days later the cells were treated as described in Fig. 2B. Seven days after treatment spheroids were dissociated to single cells and the number of living cells was determined by counting (1st generation). The number of spheroids in the control was set to 100%. (B) Thereafter, equal numbers of live cells were seeded at clonal density in low-adhesion plates resulting in 2nd-generation spheroids, which were treated again 3 days after seeding. The 2nd-generation spheroids were grown until day 7 after treatment and photographed at ×100 magnification. (C) MIA-PaCa 2 and BxPc-3 cells were treated with GTC alone or combined with quercetin or sulforaphane as indicated. Seventy-two hours later ALDH1 activity was measured with the non-toxic ALDEFLUOR assay kit and FACS analysis. The data were quantified and statistically analyzed as described in Fig. 2.

To evaluate the influence of single or combined bioactive agents to the self-renewal potential, MIA-PaCa2 cells were treated with sulforaphane, quercetin, EGCG, ECG and CG alone or in combination. Seventy-two hours later the cells were seeded in clonal density, and after additional 2 weeks the formation of colonies was analyzed (Fig. 2A). Each single substance significantly decreased the colony-forming capacity and ECG or CG had a stronger effect than EGCG. The colony formation was further reduced by the combinations quercetin/sulforaphane and sulforaphane/EGCG, whereas the combinations quercetin/ECG, quercetin/CG, SF/ECG and SF/CG had no significant effect compared to single treatments. Because each of the evaluated catechins strongly reduced the colony-forming capacity, we evaluated the total green tea catechins in their natural composition and used a purified and caffeine-free green tea extract. These green tea catechins (GTC) consisted of 588 mg catechin polyphenols/600 mg extract, from which 50% were EGCG (HPLC data are available from the manufacturer: Dr. Loges + Co. GmbH). MIA-PaCa2 and BxPc-3 cells were treated with sulforaphane, quercetin or GTC alone, or in combination. Thereafter MIA-PaCa2 cells and BxPc-3 cells were seeded for colony formation. Two weeks later, the single treatments significantly reduced colony formation, whereas the combination treatments were more effective and nearly totally abolished colony-formation (Fig. 2B). Similarly, the measurement of the self-renewal potential by the treatment of spheroidal-growing MIA-PaCa2 cells revealed a strongly reduced number of spheroidal-growing cells 48 h after single treatment, which was further reduced by combination treatment (Fig. 3A). BxPc-3 cells were not used for evaluation of spheroid formation, because this cell line is less aggressive and does not form spheroids (Table I). To evaluate if the surviving MIA-PaCa2 cells from the first treatment may be completely eliminated by a second round of treatment, we re-seeded equal amounts of surviving cells. After spheroid formation, the cells were treated again. This resulted in a nearly complete elimination of spheroidal-growing cells after single treatments and a nearly complete elimination by combination treatments (Fig. 3B). The observed effects on the self-renewal potential were confirmed by the measurement of ALDH1 activity, which is another indicator of the self-renewal capacity. After treatment of MIA-PaCa2 and BxPc-3 cells, the activity of ALDH1 was measured by the ALDEFLUOR substrate assay and FACS analysis. GTC alone reduced ALDH1 activity significantly, but the combinations had more pronounced effects (Fig. 3C). These data suggest that the self-renewal potential is strongly inhibited by treatment with sulforaphane, quercetin or GTC alone, but the combinations GTC/sulforaphane, GTC/quercetin or sulforaphane/quercetin are superior. Interestingly, the combination of the isothiocyanate with either polyphenol (GTC or quercetin) was stronger than the combination of two different polyphenolic agents.

### The combination of dietary agents is superior in reducing viability, migratory potential along with induction of apoptosis compared to single agents

**Figure 4:**
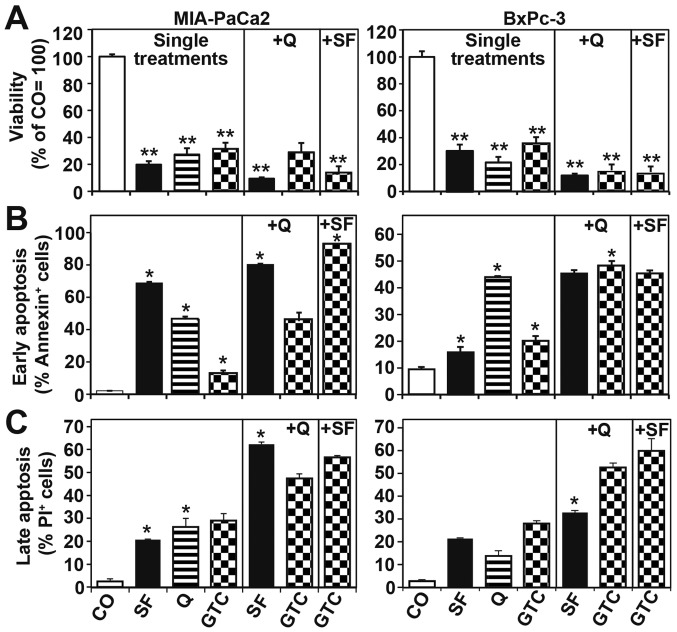
Combinations of dietary agents are superior in reducing viability and inducing apoptosis compared to single agents. (A) MIA-PaCa2 and BxPc-3 cells were treated as described in Fig. 2B. Ninty-six hours later viability was detected by the MTT assay. (B) Apoptosis was measured by annexin staining, followed by FACS analysis. The percentage of annexin-positive cells is shown. (C) Apoptosis was measured by staining of the cells with propidium iodide followed by FACS-analysis. The data were quantified and statistically analyzed as described in Fig. 2.

**Figure 5:**
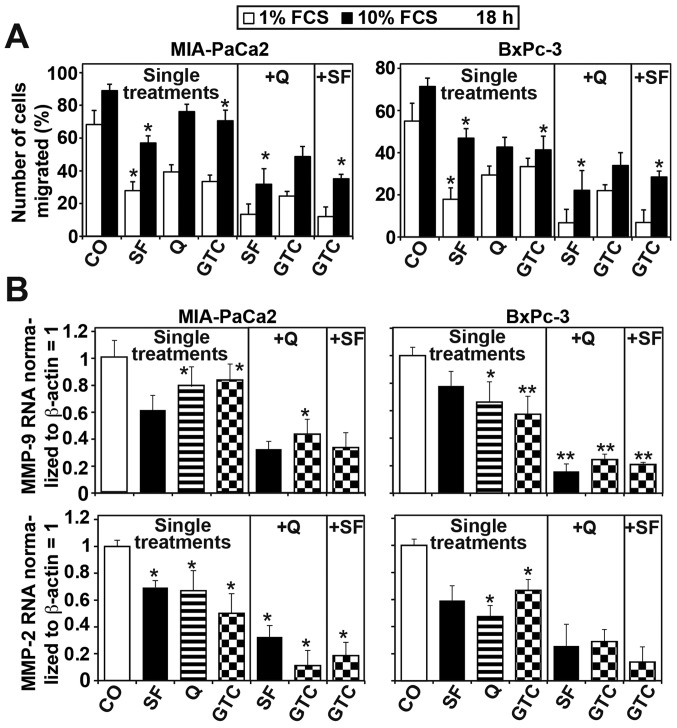
Combinations of dietary agents are superior in reducing migration and matrix metalloproteinase expression compared to single agents. (A) MIA-PaCa2 and BxPc-3 cells were treated as described in Fig. 2B. Twenty-four hours later the medium was changed to remove bioactive agents and the transmigration of cells was analyzed after additional 18 h using a standard transwell assay. The migration of cells towards medium with 10 or 1% FCS in the lower chamber was evaluated and the total number of cells in the upper chamber of the control was set to 100%. The results were quantified and statistically analyzed as described in Fig. 2. (B) MIA-PaCa2 and BxPc-3 cells were treated as described in Fig. 2B. Forty-eight hours later mRNA was harvested and the expression of matrix metalloproteinase 9 (MMP-9) or matrix metalloproteinase 2 (MMP-2) was examined by qRT-PCR. The data are shown as fold change of matrix metalloproteinase RNA levels, which were normalized to β-actin, whereas the control was set to 1. The data were quantified and statistically analyzed as described in Fig. 2.

To investigate whether GTC, sulforaphane and quercetin, alone or combined, may affect viability, apoptosis and migration potential, MIA-PaCa2 and BxPc-3 cells were treated as described above. Ninty-six hours later the NAD(P)H content was detected by the MTT assay, which reflects viability (33). Whereas all single agents strongly inhibited the viability, the combinations were stronger (Fig. 4A). Likewise, early apoptosis was induced in MIA-PaCa2 cells by single agents, but the combinations were more effective as measured by annexin staining and FACS-analysis (Fig. 4B). Comparable results were obtained by the measurement of late apoptosis by staining of the cells with propidium iodide and FACS analysis. The combination of a isothiocyanate with a polyphenol had more pronounced effects than the combination of two polyphenols, whereas all combinations were significantly stronger than the single treatments (Fig. 4C). To gain knowledge on whether the dietary agents also inhibit the migratory potential, we performed a boyden chamber assay. MIA-PaCa2 and BxPc-3 cells were treated with single agents or combinations thereof and 24 h later living cells in 10% FCS were put to the upper chamber and 1 or 10% FCS were added to the lower chamber. The transmigration of the cells was analyzed 18 h later and the number of control cells seeded to the upper chamber was set to 100%. Each single component significantly inhibited migration whereas the total effects were stronger with a gradient toward 1% FCS compared to a gradient toward 10% FCS. However, the combinations were most effective in both cell lines and with both gradients (Fig. 5A). Accordingly, the RNA expression of matrix metalloproteinase MMP-2 and MMP-9 was inhibited with strongest effects upon combination treatments (Fig. 5B). These data suggest that the combinations of quercetin, sulforaphane and GTC sensitize pancreatic cancer cells to apoptosis and inhibit their invasion potential stronger than each single agent.

### The combination of dietary agents enhances the expression of miR-let-7a associated with inhibition of K-ras stronger than the single agents

**Figure 6:**
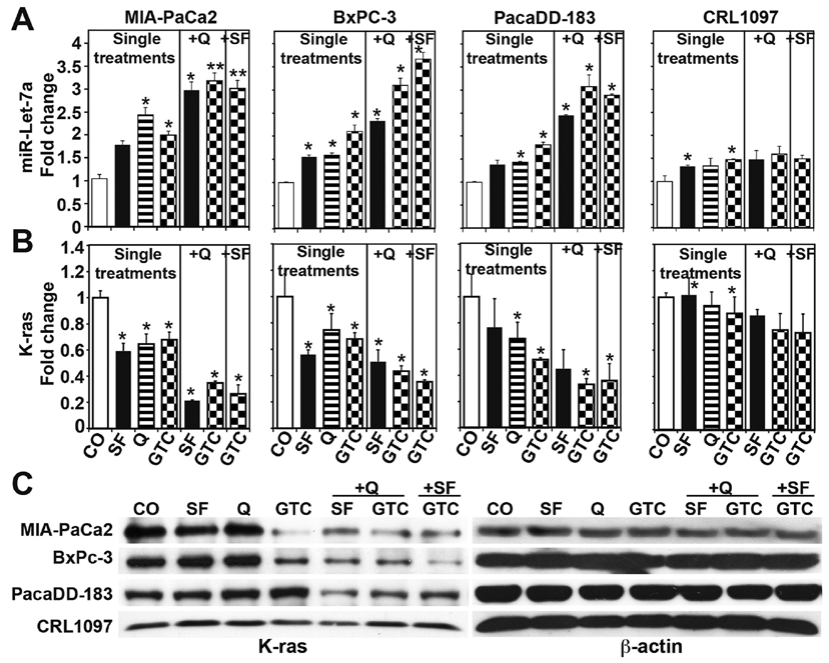
Combinations of dietary agents are superior in enhancing the expression of miR-let-7a along with inhibition of the target gene K-ras compared to single agents. MIA-PaCa2, BxPc-3, primary PDA cells (PacaDD-183) and non-malignant pancreatic ductal cells (CRL1097) were treated as described in Fig. 2B. Seventy-two hours later mRNA was harvested and the expression of (A) miR-let-7a and of (B) K-ras was examined by qRT-PCR. The data were normalized to RNU6 or β-actin for miR-let7a or K-ras, respectively, and the control was set to 1. (C) Proteins were harvested 24 h after treatment of cells and the expression of K-ras was examined by western blot analysis. The expression of β-actin served as control for equal conditions.

MIA-PaCa2, BxPC-3, primary PDA cells PacaDD-183 and CRL 1097 non-malignant pancreatic ductal cells were treated with dietary agents alone or in combination and 72 h later the expression of the miR-let-7a and its target gene K-ras was examined by qRT-PCR. The single treatments significantly enhanced the expression of miR-let-7a but inhibited the expression of K-ras in the 3 malignant cells with strongest effects after combination treatment (Fig. 6A and B). In contrast, the induction of miR-let-7a expression along with the inhibition of K-ras expression were minimal in the non-malignant CRL 1097 cells, as expected. Likewise, the basal expression of miR-let-7a was lower in malignant cells compared to non-malignant cells (data not shown). The strong inhibition of K-ras in malignant cells only was confirmed by western blot analysis (Fig. 6C). These data suggest that sulforaphane, quercetin and GTC mediate the induction of miR-let-7a expression, which in turn inhibits K-ras expression and CSC features in pancreatic cancer.

## Discussion

Considering the still devastating prognosis of patients with pancreatic cancer, we evaluated in the present study an experimental combination strategy of bioactive dietary agents for elimination of CSCs. We tested the efficacy of quercetin, sulforaphane and GTC to inhibit the self-renewal potential, apoptosis resistance and migratory potential and found that the combination of bioactive agents is more effective than the each single agents. Further, we addressed whether the induction of miR-let-7a along with inhibition of its target gene K-ras is involved. We show that sulforaphane, quercetin and EGCG strongly inhibit colony formation and demonstrate that the catechins ECG and CG are even more effective than the well examined EGCG. To mimic the natural composition of catechins in green tea, all the subsequent experiments were performed with purified green tea extract (GTC). GTC effectively inhibited the self-renewal potential, as exemplified by colony and spheroid formation as well as ALDH1 activity, but the combination with sulforaphane or quercetin increased the effect. Similar results were obtained for apoptosis resistance, migratory potential, and expression of matrix metalloproteinases. Most importantly, our data provide evidence that the combination of sulforaphane or quercetin with GTC activates the expression of miR-let-7a along with inhibition of K-ras expression much stronger than the single agents. Because upregulation of miR-let-7a and downregulation of K-ras was specific for cancer cells and occurred only minimally in nonmalignant pancreatic ductal cells, these data suggest a cancer cell-specific action of the evaluated dietary agents.

This study provides data to support the speculation, that miR-let7a-mediated inhibition of K-ras expression directly mediates the observed inhibition of CSC features. In agreement with data derived from mouse models of lung and colon cancer, oncogenic K-ras accelerated tumor progression by imposing an immature stem-like state in which the differentiation is inhibited (reviewed in ref. 34). Compelling evidence also exists for K-ras-induced reprogramming of pancreatic acinar cells into ductal intraepithelial neoplasia, a histologically well-defined precursor to PDA (34). A recent study shows the direct reprogramming of primary mouse cells by conditional expression of K-ras (34) and it was concluded that non-CSCs have the potential to dedifferentiate and acquire stem cell properties as a direct consequence of K-ras-induced plasticity. This allows the formation of CSCs with high metastatic capacity at any time during cancer progression, or vice versa, the reversal of the stem cell phenotype of existing CSCs at any time during cancer development and progression.

We found that the green tea catechins ECG and CG are more effective than EGCG in inhibition of colony formation of the highly aggressive and CSC-enriched PDA cell line MIA-PaCa2. A previous study (19) has already demonstrated that ECG and CG exert much stronger anti-proliferative and anti-inflammatory activities on the established PDA cell lines PancTu-I, Panc1, Panc89 and BxPc-3 than the most widely studied catechin EGCG. Further, our *in vitro* data are confirmed by a recent *in vivo* examination, in which EGCG inhibited the growth of orthotopically implanted human pancreatic Panc1 xenografts in BALB/c nude mice (35). Most importantly, in the latter *in vivo* study, no obvious side effects of EGCG in mice were observed. These data match our observation of a cancer cell-specific induction of miR-let- 7a and inhibition of K-ras. Our study provides data to support the hypothesis that the antitumor effect *in vivo* will be much more pronounced upon the use of the total green tea catechins combined with sulforaphane or quercetin.

We observed that the combination of the isothiocyanate sulforaphane with green tea polyphenols was similar or slightly higher in inhibition of self-renewal potential than the combination of green tea polyphenols with the polyphenol quercetin. It is tempting to speculate that this might be due to targeting of identical pathways by bioactive agents with similar chemical structures. Consequently, the combination of bioactive agent with different chemical structures may be more effective than, e.g., the combination of two different polyphenols, because a broader spectrum of anticancer pathways may be induced. The recent findings that the polyphenols resveratrol, genistein and curcumin possess anti-CSC activity (17) together with our data emphasizes that bioactive polyphenols should be combined with chemically different plant substances with anti-CSC activity to activate a wide spectrum of stem cell signaling pathways. In addition to sulforaphane and other isothiocyanates, the diterpenoid triepoxide triptolide may be a promising combination partner. Triptolide has a long history in Traditional Chinese medicine for the treatment of rheumatoid arthritis and cancer and it is derived from the vine-like herb *Tripterygium wilfordii* Hook f (36). In our prior study we demonstrated that triptolide effectively inhibits NF-κB activity, epithelial-mesenchymal transition and stem-like features in PDA cells (32).

Regarding the upregulation of miR-let-7a after treatment with sulforaphane, quercetine and green tea catechins, these data are similar to the recent notion that curcumin-induces miR-let-7a expression (20). Moreover, upregulation of miRNA-210 may contribute, which was observed after EGCG treatment and this suppressed the growth of lung cancer cells (37). As well, miRNA-30b may be involved, because it was downregulated by the treatment of HepG2 liver cancer cells with EGCG (38). Besides, the watercress-derived phenethyl-isothiocyanate downregulated miRNA-141, which repressed the expression of the androgen receptor and the PSA level in prostate cancer cell lines (39). In another study, Shan *et al* demonstrated the induction of miRNA-200c by sulforaphane in bladder cancer cells and this inhibited Cox-2, epithelial-mesenchymal transition and MMP-2 and -9 expression (40). In this respect, previous studies including our own add important information to the growing body of evidence that dietary agents may prevent cancer by epigenetic signaling. Future studies will have to address the effect of a special ‘epigenetic diet’ created for the presence of selected bioactive agents for prevention and treatment of cancer (41). Indeed, previous studies have shown that chemopreventive nutritional polyphenols and isothiocyanates may neutralize genetic defects by epigenetic regulation, which is the potential reason why these substances attenuated the processes of tumorigenesis, progression and metastasis and sensitized for drug treatment (42,43). Such epigenetic regulation does not only include the modification of miRNA expression, but may as well involve changes in histone acetylation and promoter methylation.

Together, the translational conclusion from our study is that a defined diet consisting of fruits and vegetables with experimentally proven anti-CSC activity may be a highly effective prevention of cancer growth and progression.

## Figures and Tables

**Table I:** Characteristics of the established pancreatic cancer cell lines.

CSC-properties	CSC^low^ BxPc-3	CSC^high^ MIA-PaCa2	Refs.
Source	Primary tumor	Primary tumor	ATCC
Tumor grade	G2	G3	(31)
p53 status	MT	MT	(31)
K-ras status	WT	MT	(31)
*In vitro* morphology	Densely attached	Loosely attached	(15)
Self renewal capacity			
Colony-formation	+	+++	(14,32)
Spheroid formation	−	+++	(14,32)
ALDH1 activity	+	+++	(14,32)
Gemcitabine resistance	+	+++	(14)
E-cadherin	+++	−	(18)
Vimentin	+	+++	(18)

## Abbreviations

CSC: cancer stem cell
CG: catechin gallate
ECG: epicatechin gallate
EGCG: epigallocatechin gallate
GTC: green tea catechins
PDA: pancreatic ductal adenocarcinoma
